# The validity and reliability of the Indonesian version of the Summated Xerostomia Inventory

**DOI:** 10.1111/ger.12494

**Published:** 2020-09-15

**Authors:** Yuniardini Septorini Wimardhani, Febrina Rahmayanti, Diah Ayu Maharani, Wiwik Mayanti, William Murray Thomson

**Affiliations:** ^1^ Department of Oral Medicine Faculty of Dentistry Universitas Indonesia Jakarta Indonesia; ^2^ Department of Preventive and Public Health Dentistry Faculty of Dentistry Universitas Indonesia Jakarta Indonesia; ^3^ Oral Medicine Residency Program Faculty of Dentistry Universitas Indonesia Jakarta Indonesia; ^4^ Department of Oral Sciences, Sir John Walsh Research Institute Faculty of Dentistry The University of Otago Dunedin New Zealand

**Keywords:** Indonesia, Summated Xerostomia Inventory, validity and reliability, xerostomia

## Abstract

**Objective:**

To validate and determine the reliability of the Indonesian version of the Summated Xerostomia Inventory (SXI‐ID) questionnaire.

**Background:**

Xerostomia is a common problem in older people, and the SXI is increasingly being used to measure it.

**Materials and methods:**

The SXI questionnaire was cross‐culturally adapted to create an Indonesian version (SXI‐ID), which was assessed for validity and reliability in a cross‐sectional study of older adults living in nursing homes in Jakarta, Indonesia. Each participant signed an informed consent and was interviewed with the SXI‐ID. A subset of participants was interviewed again after two weeks. A standard question was used to check criterion‐related validity, by plotting the mean SXI‐ID scale scores against the ordinal response categories of the standard question. The reliability check included Cronbach's alpha, total and inter‐item correlation, and intraclass correlation for internal consistency, along with test‐retest reliability.

**Results:**

A total of 110 older adults participated. Cronbach's alpha value for the SXI‐ID was .85, and the intraclass correlation coefficient value for test and retest in 15 participants was .9. The SXI‐ID total score showed a strong positive correlation (.87) with the global question. There was a consistent ascending gradient in mean SXI‐ID scores across the ordinal response categories of the global item.

**Conclusion:**

The SXI‐ID is psychometrically valid and reliable for measuring xerostomia in the Indonesian population.

## 1 INTRODUCTION

Dry mouth is one of the most common complaints among older people.^1^, ^2^ When dry mouth occurs, there are several consequences related to the oral cavity, including development of caries, problems with dentures and a higher risk of fungal infection among others.^3^, ^4^ Furthermore, it can influence the psychological aspects of the affected individuals, with effects such as frustration, embarrassment, unhappiness or impairment of quality of life.^3^ The two manifestations of dry mouth are salivary gland hypofunction (SGH) and xerostomia.^5^ The subjective symptom is xerostomia, while the objective sign of it is salivary gland hypofunction.^6^, ^7^, ^8^ While SGH is quantified using sialometry, xerostomia, as the subjective manifestation of dry mouth, must be measured by directly asking the individual.^1^, ^9^ That can be done using a single‐item question (“global item”) or with multiple item approaches such as batteries of items or summated rating scales.^1^

In 1999, Thomson et al^10^ described the Xerostomia Inventory (XI), which comprised 11 questions that could detect and measure the severity of xerostomia. Later, the XI was modified to a short‐form version, termed the Summated XI (SXI).^11^ The SXI uses fewer items and a simpler response format which is more suitable for frail older people. Items in the XI which were not directly related to dry mouth (such as those on dryness of the eyes, nose and facial skin), and the behavioural consequences of dry mouth were not included in the SXI questionnaire.^11^

The SXI questionnaire has been used to assess xerostomia in several different countries, and it has been adapted and validated in Dutch, English, Chinese, Portuguese and Japanese.^12^ Indonesia is the fourth most populous country, with ever‐increasing numbers of older people.^13^ To date, there has been no validation of the Indonesian version of this questionnaire. Accordingly, this study aimed to validate an Indonesian version of the SXI questionnaire to measure xerostomia in the older Indonesian population.

## 2 MATERIALS AND METHODS

### 2.1 Study design

This was a cross‐sectional study which aimed to assess the validity and reliability of the Indonesian version of the Summated Xerostomia Inventory (SXI‐ID) questionnaire among residents of government nursing homes in Jakarta, Indonesia. DKI Jakarta Province has 5 nursing homes in different areas across the province.

### 2.2 Data collection

This study was conducted in all people aged ≥60 years who had been living in a government nursing home in Jakarta for at least 1 month, who, understood Indonesian, were able to communicate well without cognitive impairment, had a Mini‐mental State Examination (MMSE) value of >24, and who provided written informed consent. Excluded were people with apraxia, terminal illness, fever or Sjögren's syndrome, and those who had undergone (or were currently undergoing) head and neck radiotherapy. This study was reviewed and approved by the Faculty of Dentistry Universitas Indonesia Research Ethics Committee (No. 82/Ethical Approval/FKGUI/X/2018).

After the study was explained to the participants, they were interviewed by a researcher who administered the MMSE questionnaire to assess the cognitive function of the participant. Another researcher then conducted the SXI‐ID interview. The interview method was adjusted considering the limitations of older people (such as low educational background and weak eyesight). Accordingly, the declarative sentences in the questionnaire were amended to become interrogative sentences. Each participant was asked to answer the SXI‐ID and was told that the answer should be the one that immediately came to their mind. The participants for the test‐retest analysis were selected from one nursing home that we visited for the first time. The participants in the retest were randomly chosen from those who had been previously assessed.

### 2.3 Questionnaire

The SXI‐ID questionnaire consists of 5 items, each of which has 5 response options: 1 = never, 2 = hardly ever, 3 = occasionally, 4 = frequently and 5 = always. There is also 1 standard dry mouth question (“How often does your mouth feel dry?”) with 4 answer options (1 = never, 2 = sometimes, 3 = often, 4 = always), which is used as a validity check.

Before testing for validity and reliability, the SXI‐ID was cross‐culturally adapted. The questionnaire was translated into Indonesian using the guidelines for cross‑cultural adaptation.^14^, ^15^ The adaptation process was undertaken according to a previously published method consisting of the 6 stages of translation, synthesis, back‐translation, expert review, testing the pre‐final version and having the adapted version to be used.^14^, ^15^ This resulted in the final questionnaire that was used for the study (Table 1).

**Table 1:** The original English version (SXI) and Indonesian version (SXI‐ID) of Summated Xerostomia Inventory

	Summated Xerostomia Inventory (SXI)	Summated Xerostomia Inventory Indonesian Version (SXI‐ID)
SXI‐1	My mouth feels dry	Apakah mulut anda terasa kering?
SXI‐2	I have difficulty in eating dry foods	Apakah anda kesulitan makan makanan yang kering?
SXI‐3	My mouth feels dry when eating a meal	Apakah mulut anda terasa kering saat sedang makan?
SXI‐4	I have difficulties swallowing certain foods	Apakah anda kesulitan menelan makanan tertentu?
SXI‐5	My lips feel dry	Apakah bibir anda terasa kering?
	Scoring: 1 = never, 2 = almost never, 3 = sometimes; 4 = often, 5 = always	1 = tidak pernah, 2 = hampir tidak pernah, 3 = kadang‐kadang, 4 = sering, 5 = selalu
Standard question	How often does your mouth feel dry?	Seberapa sering mulut anda terasa kering?
	Scoring: 1 = never, 2 = sometimes, 3 = often, 4 = always	1 = tidak pernah, 2 = kadang‐kadang, 3 = sering, 4 = selalu

### 2.4 Statistical analysis

Data were analysed using the Statistical Package for Social Sciences (SPSS) program, version 22. The SXI‐ID score was computed by summing the item response scores. We further adapted the 5‐response format to the 3‐response format, as follows: 1 = 1; 2 through 3 = 2; and 4 through 5 = 3 for statistical analysis. In the 3‐point Likert scale, 1 = never, 2 = occasionally and 3 = often. The level of statistical significance was set at *P* = .05. An item‐total correlation procedure was conducted to determine the correlation of each SXI‐ID item with the total score; each correlation value should be above 0.3. Reliability was evaluated using Cronbach's alpha. An alpha above 0.7 indicates acceptable reliability, while an alpha above 0.8 indicates good reliability.^16^ The procedures were repeated for 15 participants after 2 weeks in order to evaluate the test‐retest reliability of the SXI‐ID. The reliability was assessed using the intraclass correlation coefficient (ICC), computed using a one‐way random‐effects model.^17^ ICC values of 0.6‐0.8 indicate good reliability, whereas values above 0.8 are optimal.^18^ Convergent validity was assessed by examining the correlation between the SXI‐ID score and the standard question using Spearman's correlation coefficient. The correlation was considered poor if the coefficient value was below .20, good if the value was .41‐.60, very good if the value was .61‐.80 and near perfect if the value was above .81.^18^ Criterion‐related validity was analysed by comparing the mean total SXI‐ID scale scores across the responses to the standard question using the Kruskal‐Wallis test.

## 3 RESULTS

### 3.1 Participants

Sociodemographic information on the 110 participants is summarised in Table 2. There were 40 participants (36.3%) with a systemic condition such as hypertension, diabetes mellitus, rheumatoid arthritis, high cholesterol or cardiac disease. One or more xerogenic medications were taken by 31.8%. Furthermore, none of the participants consumed alcohol and 22.7% were currently smoking.

**Table 2:** Overview of sociodemographic and health characteristics of the participants

Characteristic	Mean (SD)	n (%)
Age	69.3 (7.6)	
Gender		
Male		58 (52.7)
Female		52 (47.3)
With systemic condition		40 (36.3)
Taking xerogenic medication		35 (31.8)
Alcohol consumption		0 (0.0)
Current smoker		25 (22.7)

### 3.2 Responses to the SXI‐ID

The responses of the study participants to each SXI‐ID item using are presented in Table 3. There were no missing SXI‐ID data. The mean total SXI‐ID score was 6.6 (SD, 2.3).

**Table 3:** Responses of study participants to each SXI item (N = 110)

SXI Item	Responses			
	Never n (%)	Occasionally n (%)	Often n (%)	Mean (SD)
SXI‐1	65 (59.1)	35 (31.8)	10 (9.1)	1.5 (0.6)
SXI‐2	89 (80.9)	17 (15.5)	4 (3.6)	1.2 (0.5)
SXI‐3	97 (88.2)	10 (9.1)	3 (2.7)	1.1 (0.4)
SXI‐4	91 (82.7)	12 (10.9)	7 (6.4)	1.2 (0.5)
SXI‐5	70 (63.6)	28 25.5)	12 (10.9)	1.4 (0.6)

### 3.3 Validity and reliability of SXI‐ID

The correlations among SXI‐ID items are shown in Table 4. The correlation between the SXI‐ID summary score and the standard question was 0.87 (95% CI 0.82 to 0.91). Cronbach's alpha was .85, indicating very good reliability. The inter‐item correlation values ranged from .40 to 1.00.

**Table 4:** Correlations among the individual SXI‐ID items and the standard question (Spearman's correlation)

	Standard question	SXI‐1	SXI‐2	SXI‐3	SXI‐4	SXI‐5	SXI‐ID
Standard question	—						
SXI‐1	1.00	—					
SXI‐2	0.58	0.58	—				
SXI‐3	0.51	0.50	0.70	—			
SXI‐4	0.60	0.59	0.82	0.82	—		
SXI‐5	0.53	0.53	0.40	0.49	0.40	—	
SXI‐ID total score	0.87	0.87	0.67	0.59	0.68	0.78	—

To assess the reliability of the SXI‐ID questionnaire, it was repeated with 15 participants after 2 weeks. The mean total score of the SXI‐ID for test and retest was both 7.0 (SD, 3.2). The ICC for test‐retest reliability was 0.9. The intraclass correlation coefficient (ICC) and item‐total correlation (ITC) values are shown in Table 5. These were found to be acceptable.

**Table 5:** The results of reliability assessment of the SXI using intraclass correlation coefficients (ICC) and item‐total correlations (ITC)

SXI	ICC	95% CI		ITC
		Min	Max	
Standard question	0.92	0.88	0.94	n/a
SXI‐1	1.00	1.00	1.00	0.83
SXI‐2	1.00	1.00	1.00	0.81
SXI‐3	0.94	0.82	0.98	0.82
SXI‐4	0.97	0.91	0.99	0.83
SXI‐5	0.96	0.90	0.99	0.69
Total Score SXI‐ID	0.99	0.98	0.98	n/a

In order to examine the criterion‐related validity, the mean SXI‐ID scale scores were compared against responses to the standard question (Table 6). There was a gradient in mean SXI‐ID score observed across the categories of the standard question, with the highest mean SXI‐ID score seen in those responding “Always,” and the lowest seen in the “Never” group. The gradient in mean scores across the categories of the standard questions was statistically significant.

**Table 6:** Criterion‐related validity comparison of the mean total SXI scores by responses to the xerostomia standard question

Standard question	Mean (SD) Total SXI‐ID score
Never	5.2 (0.4)
Occasionally	7.4 (1.6)
Frequently	10.4 (1.9)
Always	13.6 (2.1)

Note

(Kruskal‐Wallis test; *P* < .05).

## 4 DISCUSSION

This study has investigated and confirmed that the Indonesian version of Summated Xerostomia Inventory (SXI‐ID) has good validity and reliability for measuring xerostomia in the older Indonesian population and that it is as valid and reliable as the original English version with the 5‐point Likert scale response for each item. We aim to have a more comprehensive answer for this initial study.

This was an initial validation study, conducted with older residents of a government nursing home in Jakarta. A limitation of this study is that the findings may not be generalisable. Future use of the questionnaire in more diverse older Indonesian samples would be useful and informative. We also did not conduct a formal sample size calculation prior to conducting the study, but we did undertake a post hoc power calculation (using G*Power version 3.1.9.4) based on the collected data. This showed that our N was more than sufficient, with, for example, 10 people required to demonstrate even the difference in mean SXI score between those responding “Never” and those responding “Occasionally” to the standard question (given the observed effect size of 1.0 and the power of 0.95 to demonstrate a difference).

Despite the limited number and diversity of participants, a strength of this study was the cognitive function screening of the participants using the MMSE. This ensured that participants were cognitively able to respond to the SXI‐ID, and so there were no missing data. Only older adults with an MMSE score >24 were included. We further formatted the responses into a 3‐point Likert scale format for consistency with previous studies.^19^

The construct and discriminant validity of the SXI‐ID questionnaire was tested through associations and comparisons between the scores of the SXI‐ID and the standard question. The mean Cronbach's alpha value of .90 suggests that the questions in the SXI‐ID are measuring same construct and have excellent internal consistency. The data indicate that the SXI‐ID questionnaire is valid for use as a measuring instrument in Indonesia. The mode of questionnaire administration may have influenced the validity of the findings. In this study, the data collection from participants used a standard interview approach with the SXI‐ID items as interrogative statements without adding any information to the interview. There was only one interviewer, to eliminate inter‐interviewer bias. In this study, interviewing was chosen as the method of administration over the self‐administered method in order to maximise participation and minimise cognitive burden bias.^20^ The original SXI validation^11^ used six different geriatric population samples; half of those studies used interviews, and half used written questionnaires. Comparison of the psychometric and criterion validation data shows no systematic differences (tables II and III, and figure 1 in that paper), suggesting that our choice of administration method in the current study is unlikely to have unduly affected the findings.

The correlations observed in this study were well above the recommended threshold and were similar to those obtained using Chinese and Portuguese versions of the SXI.^18^, ^19^, ^21^

The current study also showed the expected gradient in mean SXI‐ID scores across the response categories of the standard question, suggesting that the SXI‐ID meets the criteria for independent validation. Exploration of its association with objective measures of salivary flow rate is needed, although of course the SXI‐ID is not measuring salivary flow. Further investigation of the SXI‐ID in studies using sialometry would be informative.

There has been little investigation of the test‐retest reliability of the original XI and SXI in previous studies. Our findings provide important evidence for the instrument's test‐retest reliability.

The SXI has now been successfully adapted and validated for use in Indonesia. Accordingly, it can now be used in both the clinical and epidemiological settings. The Summated Xerostomia Inventory Indonesia Version (SXI‐ID) has the potential to be used for millions of Indonesians, in order to help Indonesia to face the increasing numbers of older people or people with medical conditions (multimorbidity and polypharmacy) who might have xerostomia. Monitoring the severity of dry mouth complaints using this Questionnaire would be important for their ongoing health care.^22^, ^23^

## 5 CONCLUSION

The SXI‐ID has excellent psychometric properties and is a valid and reliable tool for measuring xerostomia in the Indonesian population, much like its parent English version.

## AUTHOR CONTRIBUTIONS

YSW planned and assisted the survey, supervised the study, drafting and finalising the manuscript. FR dealt with local bureaucracy, obtained study permits, acted as a liaison to obtain ethical clearance, and supervised the study and drafting of the manuscript. DAM contributed to statistical analysis and data interpretation. WM contributed to data collection, data analysis and initial data report. WMT revised the manuscript critically for important intellectual content and contributed to the drafting and finalising of the manuscript. All authors have approved the final version and agreed to be accountable for all aspects of the work.
